# Mechanosensitivity in Pulmonary Circulation: Pathophysiological Relevance of Stretch-Activated Channels in Pulmonary Hypertension

**DOI:** 10.3390/biom11091389

**Published:** 2021-09-21

**Authors:** Solène Barbeau, Guillaume Gilbert, Guillaume Cardouat, Isabelle Baudrimont, Véronique Freund-Michel, Christelle Guibert, Roger Marthan, Pierre Vacher, Jean-François Quignard, Thomas Ducret

**Affiliations:** 1 Centre de Recherche Cardio-Thoracique de Bordeaux, Univ. Bordeaux, U1045, F-33600 Pessac, France; solene.barbeau@u-bordeaux.fr (S.B.); guillaume.cardouat@u-bordeaux.fr (G.C.); isabelle.baudrimont@u-bordeaux.fr (I.B.); veronique.michel@u-bordeaux.fr (V.F.-M.); christelle.guibert@u-bordeaux.fr (C.G.); roger.marthan@u-bordeaux.fr (R.M.); pierre.vacher@inserm.fr (P.V.); jean-francois.quignard@u-bordeaux.fr (J.-F.Q.); 2 INSERM, Centre de Recherche Cardio-Thoracique de Bordeaux, U1045, F-33600 Pessac, France; 3 ORPHY, UFR Sciences et Techniques, University of Brest, EA 4324, F-29238 Brest, France; guillaume.gilbert@univ-brest.fr

**Keywords:** calcium, endothelial cell, fibroblast, mechanosensitive channel, pulmonary arterial smooth muscle cell, pulmonary artery, pulmonary hypertension, Piezo channel, TRP channel, vascular cell

## Abstract

A variety of cell types in pulmonary arteries (endothelial cells, fibroblasts, and smooth muscle cells) are continuously exposed to mechanical stimulations such as shear stress and pulsatile blood pressure, which are altered under conditions of pulmonary hypertension (PH). Most functions of such vascular cells (e.g., contraction, migration, proliferation, production of extracellular matrix proteins, etc.) depend on a key event, i.e., the increase in intracellular calcium concentration ([Ca^2+^]_i_) which results from an influx of extracellular Ca^2+^ and/or a release of intracellular stored Ca^2+^. Calcium entry from the extracellular space is a major step in the elevation of [Ca^2+^]_i_, involving a variety of plasmalemmal Ca^2+^ channels including the superfamily of stretch-activated channels (SAC). A common characteristic of SAC is that their gating depends on membrane stretch. In general, SAC are non-selective Ca^2+^-permeable cation channels, including proteins of the TRP (Transient Receptor Potential) and Piezo channel superfamily. As membrane mechano-transducers, SAC convert physical forces into biological signals and hence into a cell response. Consequently, SAC play a major role in pulmonary arterial calcium homeostasis and, thus, appear as potential novel drug targets for a better management of PH.

## 1. Introduction

**Figure 1:**
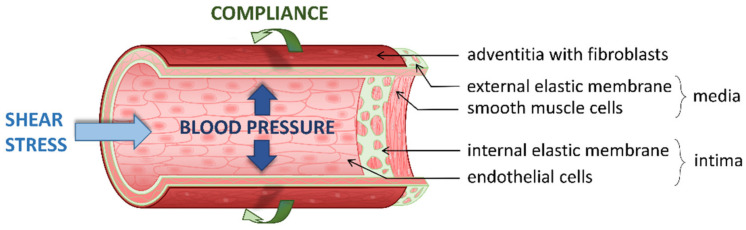
Hemodynamic forces acting on the vessel wall. Section of an artery wall showing that the endothelial cells, forming the inner tunica, are longitudinally aligned, whereas smooth muscle cells, forming the median layer, are circumferentially aligned; the surrounding adventitia predominantly includes fibroblasts and matrix. Shear stress, frictional force generated by blood flow, is parallel to the vessel wall, whereas blood pressure is perpendicular to the vessel wall, causing circumferential and longitudinal stretching. Beside blood mechanical forces, composition of extracellular matrix, contributing to arterial stiffness, may itself modulate compliance and mechanotransduction in the vessel wall.

Pulmonary arteries (PA) are continually subjected to mechanical forces exerted by circulating blood on the three-layered vessel wall (i.e., intima, media, and adventitia) (Figure 1). Among these forces, shear stress and pulsatile blood pressure are the most notable (for review see [1]). Shear stress, the frictional force generated by blood flow, is parallel to the vessel wall and mainly acts on pulmonary arterial endothelial cells (PAEC) which are longitudinally aligned and form the inner tunica of the vessels. Conversely, blood pressure is a perpendicular force that causes a circumferential and a longitudinal stretch to the vessel wall, proportionally to the cardiac output resistance. Blood pressure acts both on pulmonary arterial smooth muscle cells (PASMC) which align circumferentially and form the median layer, and, to a lesser extent, on PAEC.

Shear stress and blood pressure have opposite effects: the former, acting on PAEC, induces a vasodilatation, whereas the latter, acting on PASMC, exerts an intrinsic vasomotor mechanism termed “myogenic tone” [2]. However, these two phenomena are conveyed through an elevation of the intracellular Ca^2+^ concentration ([Ca^2+^]_i_) that involves the superfamily of stretch-activated channels (SAC) and acts as an intermediate to physiological responses. Indeed, shear stress increases the [Ca^2+^]_i_ in PAEC leading to a vasodilatation via, particularly, nitric oxide (NO) production that, in turn, influences adjacent PASMC contraction. Intravascular pressure, acting on PASMC, is also directly transduced by SAC, which can serve as direct route for Ca^2+^ entry and/or cause membrane depolarization which secondarily activates voltage-dependent Ca^2+^ channels. The resulting [Ca^2+^]_i_ increase subsequently activates PASMC contraction through activation of calmodulin and myosin light chain kinase.

Beside blood mechanical forces, the composition of the extracellular matrix (ECM) also contributes to arterial stiffness and may, *per se*, modulate mechanotransduction in the vessel wall. Indeed, there is a complex crosstalk between ECM and vascular cells: PASMC and pulmonary artery adventitial fibroblasts (PAAF) produce the ECM, which in turn interacts with cells through SAC and different receptors such as integrins, altering stiffness-dependent vascular cell activation [3].

Mechanical stretch affects all of the main cell types (i.e., PAEC, PASMC, and PAAF) of the vessel wall and adapts the vessel tone to pressure in physiological circumstances. An alteration of mechanical stimuli is observed under pathological conditions, such as pulmonary hypertension (PH). Indeed, PH is a group of multifactorial pathological cardiovascular disorders characterized by a progressive elevation of pulmonary arterial pressure leading to right ventricular hypertrophy, heart failure and, ultimately, to premature death [4]. The recent haemodynamic definition of PH states that pre-capillary pulmonary hypertension due to pulmonary vascular disease is diagnosed when mean pulmonary arterial pressure of over 20 mmHg is associated with abnormal pulmonary vascular resistance of 3 or more Wood Units [5]. PH is divided into five groups according to clinical, hemodynamic, etiological characteristics, and treatment strategy: idiopathic and heritable pulmonary arterial hypertension (PAH) (group 1), PH due to left heart disease (group 2), PH due to lung diseases and/or hypoxia (group 3), PH associated to chronic thromboembolism (group 4), and finally PH forms with unclear or multifaceted origins (group 5) [4].

This is accompanied by an alteration of mechanical stresses to the vessel wall: an increased myogenic tone [6], a reduced flow rate [7] that is responsible for a lower shear stress [8], and a progressive PA stiffening [3]. In case of endothelium injury, occurring in PH or PA catheterization, especially during the measurement of pulmonary arterial wedge pressure, PASMC can also be directly exposed to flow shear stress or elevated transmural flow shear stress [9].

Although many mechanosensitive cellular components and extracellular structures have been shown to contribute to mechanotransduction [10], in PA wall in particular, the scope of this review is deliberately limited to SAC activation.

## 2. Stretch-Activated Channels

Plasma membrane SAC are referred to as mechanotransducers since they convert physical forces into biological signals and hence into an adaptive cell response. In the late 1970s, their existence was postulated by Corey and Hudspeth [11], before being electrophysiologically characterized, a few years later, in embryonic chick skeletal muscle by Guharay and Sachs [12]. Since their original characterization, SAC have been identified in a variety of tissues and species including mammalian vascular cells (for review see [13,14,15]) and pulmonary vascular cells in particular [9,16,17,18,19,20,21,22,23,24,25,26,27].

A common characteristic of SAC is that their open probability increases with the applied pressure, i.e., that their gating depends on membrane stretch. In pulmonary vascular cells, Ca^2+^ permeable non-selective cationic SAC share similar general electrophysiological properties: I-V relationship of the evoked currents is almost linear with a reversal potential around 0 mV and a unitary conductance around 30 pS [17,18,19,22,24]. Unlike voltage-gated channels, very few pharmacological inhibitors are available for SAC. The only specific one is GsMTx-4, a peptide toxin isolated from tarantula venom [28], which has shown inhibitory effect on SAC in PAEC [26] and PASMC [19,20,21,22,24,25]. GsMTx-4 does not act on a standard ‘‘lock and key’’ model, but acts by its incorporation into boundary lipids surrounding the channel in a tension-dependent manner and thereby reducing the transfer of force from the lipid bilayer to the channel [28]. In the context of PH, most SAC inhibitors used in experimental studies are unselective blockers: amiloride and its analogs, aminoglycoside antibiotics such as streptomycin [19,21,26], disulfonic stilbene DIDS (4,4’-diisothiocyanatostilbene-2,2’-disulfonate) [17,22], and Gd^3+^ [17,19,21,25,26]. Their non-selectivity and potential side effects certainly explain the absence of clinical studies.

**Figure 2:**
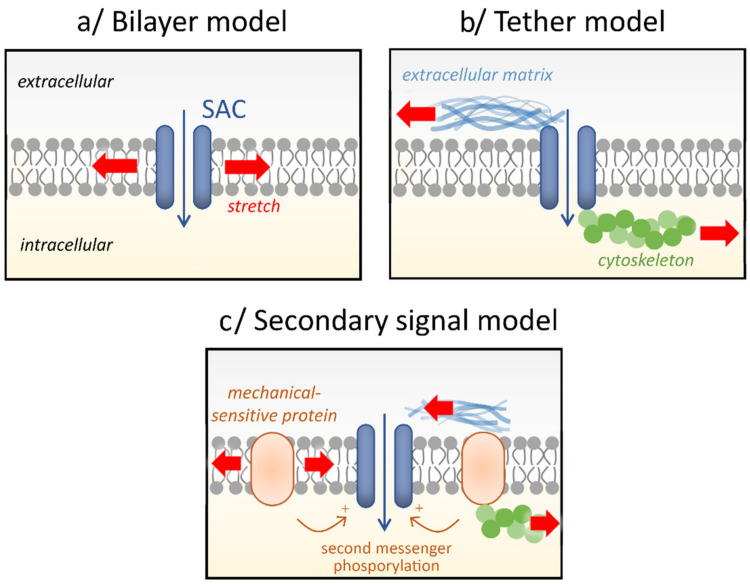
Activation mechanisms of SAC. Three general models are proposed: (**a**) In the “bilayer model”, the tension developed (red arrow) in the lipid bilayer itself is directly responsible for channel gating. (**b**) In the “tether model”, the force is transmitted to the channel via proteins located in the extracellular matrix, the cytoskeleton, or both. Tensions are conveyed by these accessory proteins to induce the channel opening. (**c**) In the “secondary signal model”, the channel activation depends on a distant mechanical-sensitive protein generating diffusible second messenger or channel phosphorylation.

As reviewed in [1], three general models are proposed to account for the activation mechanisms of SAC: the “bilayer model”, the “tether model”, and the “secondary signal model” (Figure 2).

In the “bilayer model” (Figure 2a), the tension developed in the lipid bilayer itself is directly responsible for channel gating. Different mechanisms are also described for this model in which intrinsic mechanosensitivity of the channel depends on dimensional changes and/or hydrophobic mismatch (for review see [29]). This direct mechanical activation is supported by solubilization and liposome reconstitution experiments in which SAC activity was maintained, as well as the kinetic and the reversibility of the current elicited by stretch. In the “tether model” (Figure 2b), the force is transmitted to the channel via proteins located in the extracellular matrix or the cytoskeleton or both. Thus, as demonstrated by using agents that disrupt (such as cytochalasin and colchicine) or stabilize (such as phalloidin) cytoskeleton or extracellular matrix organization, SAC opening depends on the displacement of these proteins relative to the channel. In the “secondary signal model” (Figure 2c), a distant mechanical-sensitive protein (e.g., phospholipase A2 or phospholipase C (PLC)) can release a diffusible second messenger (such as arachidonic acid and diacylglycerol (DAG)) or activate a kinase (such as protein kinase C (PKC)), which, in turn can activate the channel. These different types of activation mechanisms could exist in pulmonary vascular cells. Indeed, in PAEC and PASMC, a direct stretch activation of SAC (“bilayer” and “tether” models) is supported by the fact that the stretch-induced current is turned on and off without a noticeable delay [17,19,21,26]. Furthermore, SAC currents were recorded in inside-out patch-clamp configuration, excluding the possible involvement of secondary messengers, at the exception of enzyme-linked channels [17]. In contrast, some studies suggest an indirect mechanical activation (“secondary signal model”) in which stretch leads to activation of PKC (via stimulation of PLC and production of DAG), which, in turn, activates SAC [17,24]. Moreover, these two activation modes most likely trigger converging pathways since numerous SAC are polymodal channels (see sections below).

**Figure 3:**
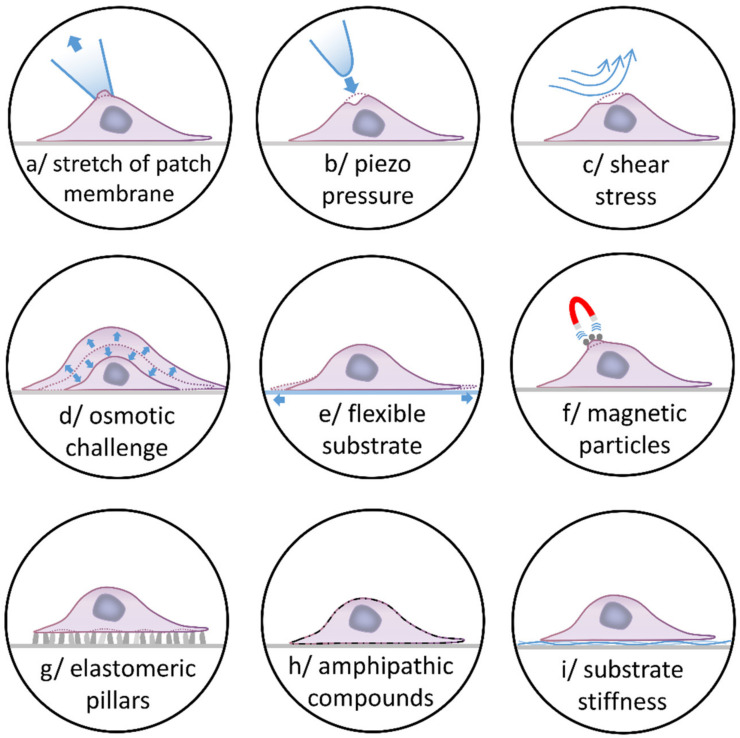
Experimental strategies to investigate SAC in cells. At the cellular level, several strategies can be used to activate SAC. The most commonly used are based on membrane deformation: (**a**) applying positive or negative pressure to the back end of the patch pipette, (**b**) poking of the cell membrane by a piezo-driven glass pipette, (**c**) modifying the perfusion flow or the viscosity of the solution, (**d**) using osmotic challenges: hypotonicity induces cell swelling, whilst hypertonicity evokes cell-shrinkage, (**e**) elongating thin elastic silicone membrane where cells are seeded, (**f**) applying magnetic field to specific ligands coated with magnetic particles on the cells, (**g**) seeding cells on elastomeric pillars to apply force to specific parts of the cells, and (**h**) using crenators and cup formers (amphipathic compounds) to induce crenation or cup shapes. (**i**) Another alternative consists in culturing cells in matrices of different stiffness, to evaluate the impact of the environment matrix and more especially its stiffness.

**Figure 4:**
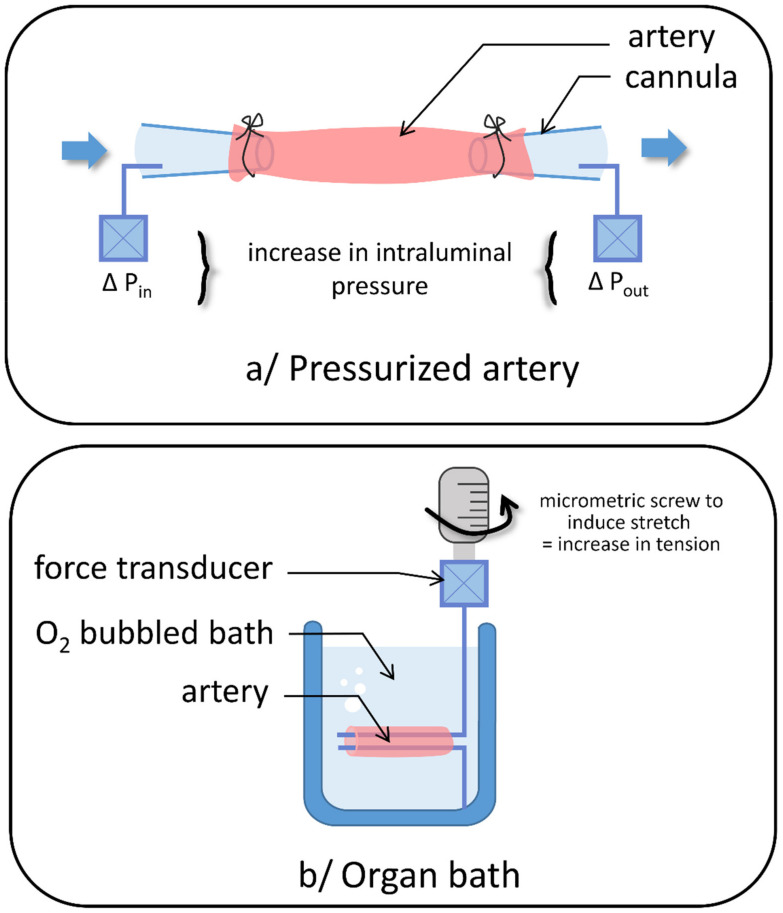
Experimental strategies to investigate SAC in vessels. At the tissue level, the effects of stretch can also be studied in whole vessels using (**a**) arteriography: the microvessel is cannulated at both ends with glass micropipettes and placed in a microvascular flow system chamber, allowing intraluminal pressure increase via modulation of inlet and outlet pressures (P_in_ and P_out,_ respectively); or (**b**) myography: one end of the segment is anchored to a stationary support and the other end is connected to a force-displacement transducer to monitor the vessel contraction under resting tension corresponding to an adapted transmural pressure.

At the cellular and tissue levels, several strategies have been developed to investigate SAC. Among cell-based assays (Figure 3), the most commonly used method is based on membrane deformation including amphipathic compounds, elastomeric pillars, elongation of flexible cell substrates, magnetic particles, osmotic challenges, patch membrane stretch, piezo-driven pressure, and shear stress (for review see [30] or [31]). These techniques have been applied to pulmonary vascular cells in vitro [9,16,17,18,19,20,21,22,23,24,25,26,27,32]. An alternative method consists in culturing cells in matrices of different stiffness in order to evaluate the impact of the environment matrix on the cells. To this end, PAAF were cultured in different stiffness polyacrylamide gels corresponding to a normotensive pulmonary artery (0.5 kPa) or mimicking mild-severe PH (3-10 kPa) [33]. Furthermore, the effect of stretch can also be studied in whole vessels for a more integrative approach using arteriography or myography (Figure 4). In the former, a microvessel is cannulated at both ends with glass micropipettes and placed in a microvascular flow system chamber, allowing intraluminal pressure increase. In the latter, one end of the segment is anchored to a stationary support and the other end is connected to a force-displacement transducer to monitor the vessel contraction under resting tension corresponding to an adapted transmural pressure. These protocols are commonly used to investigate myogenic tone [6,19,23] or basal stretch [22] in small isolated intralobar PA. Mechano-dependent contraction of vessels can also be induced by hypo-osmotic shocks [25].

As the increase in [Ca^2+^]_i_ due to activation of Ca^2+^ channels plays a key role in fundamental cellular processes involved in the pathogenesis of PH such as contraction, migration, and proliferation, and thereby in vascular remodeling and hyperreactivity, PH is often referred to as a vascular channelopathy. Thus, in the pulmonary vasculature, which is continually subjected to mechanical forces, it is likely that facilitation of Ca^2+^ entry via SAC contributes to development and/or maintenance of the pathology. Indeed, in PH of group 3, hypoxia exposure could initiate, via the hypoxic pulmonary vasoconstriction phenomenon [34], vascular tone elevation and subsequent SAC activation, contributing to the early development of PH. In other PH groups, although not being the trigger, SAC activation following alteration of mechanical stresses to the vessel could contribute to the progression and/or maintenance of the pathology. Interestingly, SAC are more active in PASMC from chronically hypoxic (CH) or monocrotaline (MCT) rats, two animal models of PH, than in those from healthy rats [19,20,25]. Moreover, in the pulmonary vasculature of normoxic rats, myogenic tone, sensitive to SAC blockers, is minimal as compared to that in the pulmonary vasculature of CH rats [6,19,23]. In addition, hypoxia exposure induces immediate upregulation of mechanosensitive TRPV4 channel in rat PA [23]. Thus, SAC appear to play a major role in PA pathophysiological signaling. There is a growing body of evidence implicating members of the Transient Receptor Potential (TRP), Piezo and other families of ion channels in this mechanotransduction, which will be described in the following sections.

## 3. TRP Channels

TRP channels constitute a superfamily of non-selective cationic channels (Table 1) that exhibit a common structure composed of N- and C-terminal regions containing protein interaction motifs and six transmembrane domains (TM1-TM6). The putative ion conducting pore is located between the 5th and 6th TM domain. TRP channels are polymodal and, hence, can be modulated by a wide variety of stimuli such as cold, heat, pH, membrane potential, mechanical stress, neurohormonal signals, Ca^2+^ and Mg^2+^ ions, oxidative stress, intracellular ligands, as well as vasoactive factors such as angiotensin-II, arachidonic acid and its metabolites epoxyeicosatrienoic acids (EET), adenosine triphosphate (ATP), thrombin, endothelin-1 (ET-1), and serotonin (5-HT). According to their activation stimuli and the presence of regulatory domains on the cytosolic N- and C-termini, TRP superfamily is subdivided into seven main subfamilies: TRPC1-7 (Canonical), TRPV1-6 (Vanilloid), TRPM1-8 (Melastatin), TRPP1-4 (Polycystin), TRPML1-3 (Mucolipin), TRPA1 (Ankyrin), and TRPN (no mechanoreceptor potential C, NOMPC). Functional TRP are composed of four homo- or heteromultimeric subunits. These latter display conduction properties and regulatory mechanisms that are distinct from those of homomeric channels. TRP channels are expressed in a wide range of cell types and, as Ca^2+^ permeable channels, they are central actors in cellular Ca^2+^ homeostasis.

Although the relevance in mechanotransduction has not been examined for many of TRP channel isoforms playing important physiological functions in pulmonary vessels, their mechanosensitivity in other tissues has been demonstrated, suggesting their putative role as SAC in pulmonary vessels. Thus, we describe in this section their role in pulmonary vasculature and involvement in PH.

### 3.1. TRPC

Among TRPC1-7 channels, TRPC1, TRPC3, TRPC4, TRPC5, and TRPC6 are sensitive to mechanical stimulation [36]. Interestingly, these five isoforms are detected in PA from different species including rat or human, and some of them play a role in PH [67,68].

#### 3.1.1. TRPC1

TRPC1 was the first member of this channel superfamily to be identified in drosophila, in 1995 [69]. This channel is expressed ubiquitously and can interact with some other TRP channels such as TRPC4, TRPC5, TRPC6, TRPP2, or TRPV4 [70]. TRPC1 is a mechanosensitive channel involved in store-operated calcium entry (SOCE) and receptor-operated calcium entry (ROCE) in various cell types playing a role in cell proliferation and migration [36,37].

In the pulmonary vasculature, previous studies have demonstrated that TRPC1 is expressed in PASMC and PAEC in rat, mouse, and human [68,71,72]. The role of TRPC1 in PA has mostly been studied under pathological condition of PH where it participates in the worsening phenotype of the disease. TRPC1 expression is upregulated at mRNA and protein levels in PA from PH animal models such as CH [68], MCT [73] or chronic ligation of the left main PA (thromboembolic PH) [74]. In PASMC from PH rat, TRPC1 is involved in basal increase in [Ca^2+^]_i_ and SOCE currents [68,74,75]. This dysregulation of Ca^2+^ homeostasis increases PASMC proliferation, observed in PH [71], and promotes PA remodeling and altered contraction responses [73,76]. Moreover, Bone Morphogenetic Protein 4 (BMP4), which plays an important role in PA remodeling, increases SOCE currents through TRPC1 overexpression in PASMC. This process is dependent on p38-ERK1/2 MAPK pathway [77,78]. In a pre-clinical study using a CH-induced PH murine model, Sun et al. demonstrated that intratracheal TRPC1 siRNA delivery reduces the production of pro-fibrosis factors, increases endothelial nitric oxide synthase (eNOS) expression that favors vasodilatation, and decreases pro-apoptotic factors, overall attenuating PH phenotype [79]. In accordance with this study, pulmonary vasculature remodeling in response to CH is reduced in *trpc1*^−/−^ mice [71]. Moreover, vasomotor tone and hypoxia-enhanced 5-HT vasoconstriction are also attenuated in these mice [76]. Furthermore, right ventricular hypertrophy and microvessels remodeling are suppressed in double knock out mice *trpc1*^−/−^/*trpc6*^−/−^. Finally, sildenafil, a phosphodiesterase-5 (PDE5) inhibitor currently used to treat PH, reduces TRPC1 expression, diminishing basal [Ca^2+^] and SOCE currents in PASMC and, consequently, the severity of PH in a CH rat model [75,80]. Overall, these findings indicate that TRPC1 plays a central role in Ca^2+^ homeostasis in PH and that regulating its expression and/or function can be used as potential therapeutic means in the disease.

#### 3.1.2. TRPC3

TRPC3 is a mechanosensitive channel ubiquitously expressed, particularly in central nervous and cardiovascular systems. TRPC3 can interact with some other TRP channels such as TRPC1, TRPC6, or TRPC7 [70]. TRPC3 is involved in SOCE and ROCE playing various roles in cardiovascular diseases [38].

TRPC3 is expressed in human and rat PA [81,82], in both PASMC and PAEC [38]. Moreover, this channel is upregulated in PASMC from patients with pulmonary arterial hypertension (PAH), at mRNA and protein levels [50]. This increase is associated with an increased SOCE [83]. However, to our knowledge, few studies reported a distinct role for TRPC3 in pulmonary vasculature or in PH.

#### 3.1.3. TRPC4

TRPC4 is a ubiquitously expressed channel involved in ROCE/SOCE currents. This channel is one of the main TRPC channels expressed in human, mouse, and rat PA. Although stretch-induced TRPC4 activation has not been shown to be involved in PH, it can be activated by a variety of stimuli including arachidonic acid, whose signaling pathway is implicated in mechanosensitivity [42,43].

In smooth muscle and endothelial cells, TRPC4 plays an important role in regulating microvascular permeability, agonist-dependent vasorelaxation, and gene transcription [67]. At cellular level, TRPC4 is involved in Ca^2+^ signaling, altering endothelial permeability in PAEC [44,47]. In pulmonary vasculature, TRPC4 promotes pulmonary arterial constriction and remodeling in rat SUGEN-Hypoxia PH group 1 model. Thus, *trpc4^−/−^* rats present attenuated PA remodeling, reduction of plexiform lesions, associated with an increase of survival [46,47].

#### 3.1.4. TRPC5

Initially identified in the central nervous system, TRPC5 is a Ca^2+^ permeable non-selective channel expressed in a range of tissues and cell types. Its expression in the pulmonary vasculature is conflicting. In some studies, this channel was detected in PASMC and PAEC [48] whereas in others, it was not detected in distal PA or PASMC [49] or detected only in PASMC but not in PAEC [50]. Despite the fact that TRPC5 role is well documented in the systemic vasculature, particularly in pathophysiological regulation of vascular tone [60,84] or in angiogenesis [85], to our knowledge, no studies have reported a distinct role for this mechanosensitive channel in PA or PH.

#### 3.1.5. TRPC6

TRPC6 is a mechanosensitive channel ubiquitously expressed in vasculature. There is some evidence that TRPC6 is involved in SOCE and ROCE, playing a role in numerous biological processes including cell proliferation, myogenic tone, and agonist-induced vasoconstriction modulation [37]. Moreover, TRPC6 is also directly activated by DAG.

In pulmonary vasculature, studies have demonstrated TRPC6 expression in PASMC and PAEC in mouse, rat, and human [86,87], in proximal and distal PA [48,49,82,88]. Moreover, TRPC6 is also present in pulmonary venous smooth muscle [89]. Interestingly, it plays various roles in PH. Firstly, TRPC6 expression is upregulated in human PA from PH patients [50] and PASMC from idiopathic pulmonary arterial hypertension (IPAH) patients [90], as well as in PH animal models such as CH [68] and MCT [91]. A polymorphism in TRPC6 is also associated with an increased risk to develop IPAH [92]. Secondly, in PASMC, TRPC6 promotes SOCE and ROCE currents, and PASMC proliferation [50,90,93]. Furthermore, TRPC6 has been implicated in the adaptive contraction response of pulmonary vasculature to alveolar hypoxia [87]. Thirdly, *trpc6*^−/−^ mice present less pulmonary vasculature remodeling in response to CH associated with a decreased vasomotor tone and hypoxia-enhanced 5-HT vasoconstriction response, attenuating the pulmonary hypertensive phenotype [76,94]. In addition, bosentan, an endothelin receptor inhibitor currently used to treat PAH, reduces TRPC6 expression, inhibiting cell proliferation [90]. Finally, as for TRPC1, sildenafil reduces TRPC6-evoked SOCE current in PASMC and consequently the severity of PH in CH rat model [75,80].

Promising new inhibitor candidates have been recently studied for PH treatment. Indeed, in CH or MCT rat models, chrysin, a component of medicinal plants, inhibits PH-induced TRPC1/6 upregulation, modulates Ca^2+^ homeostasis, reduces PASMC proliferation, and reactive oxygen species production, attenuating the PH phenotype (right ventricular pressure and hypertrophy, and vascular remodeling) [95,96]. In the same way, in CH mouse model, chloroquine, used for antimalarial treatment in Plasmodium vivax malaria, reverses TRPC1/6 overexpression and decreases vascular remodeling [97]. Finally, in CH rat model, topotecan, a toposisomerase inhibitor already used for cancer therapy, inhibits CH-induced TRPC1/4/6 overexpression and reduces [Ca^2+^]_i_ in PASMC, improving hemodynamic parameters and attenuating artery remodeling and right ventricle hypertrophy [98].

### 3.2. TRPV

Among TRPV channels, TRPV1 [99,100], TRPV2 [101], and TRPV4 [66,102,103] are known to be mechanosensitive [104], especially to an osmotic stimulation [105].

#### 3.2.1. TRPV1

TRPV1 is expressed in PAEC and PASMC [21,106,107,108,109], as well as in rat distal pulmonary venous smooth muscle cells (SMC) [110]. This channel is permeable to Ca^2+^ and contributes, to a large extent, to many Ca^2+^-signaling pathways including proliferation and migration associated with PH [21,107].

To study the functional implication of TRPV1 in PASMC, a specific agonist, capsaicin, has been used to induce Ca^2+^ responses [21,64,107]. In these cells, TRPV1 activation promotes PASMC migration associated with specific changes in the cytoskeleton architecture such as reorganization of F-actin, tubulin, and intermediate filament networks as well as NFAT nuclear translocation [21,107]. Thus, TRPV1 could activate Ca^2+^/calcineurin/NFAT cascade signaling pathway known to play a crucial role in the physiopathology of PASMC, implicated in hypoxia-induced PA remodeling and proliferation [21]. TRPV1 mRNA and proteins are increased in human PASMC exposed to CH, an in vitro model of PH [108]. Consistent with these observations, both capsaicin-induced increase of [Ca^2+^]_i_ and channel activity are enhanced in PASMC from IPAH patients [64]. On the contrary, inhibition of TRPV1 by means of antagonists or knockdown reduces human hypoxia-induced PH [108] and IPAH PASMC proliferation [64]. Another evidence of TRPV1 role in proliferation is the response of rat PASMC to acute exposure to silicium dioxide nanoparticles. After such an exposure, the increase of the [Ca^2+^]_i_ and a proliferative response are greater in PASMC isolated from rats with PH than in those from control rats, and these responses are inhibited in the presence of TRPV1 antagonist [111]. Although TRPV1 expression is not modified in rat PASMC cultured under hypoxic conditions, it seems that hypoxia induces TRPV1 membrane translocation [21]. Likewise, hypoxia exposure does not potentiate the TRPV1-induced cytoskeleton reorganization but TRPV1 inhibition limits the effect of hypoxia on cytoskeletal changes suggesting an implication of TRPV1 in this remodeling [21]. More recently, a beneficial effect of TRPV1 in PH was demonstrated through TRPV1 involvement in human PA vasodilatation in response to cannabidiol [106]. This phenomenon could be explained by the expression of TRPV1 in PAEC. Given that most of the previous studies describing the implication of TRPV1 in proliferation and migration associated with PH were performed on isolated PASMC, the signaling pathways implicated in PAEC after TRPV1 activation could provide counterbalancing effects at the site of the entire vessel.

#### 3.2.2. TRPV2

Although TRPV2 is expressed in PA, its function has been less studied. mRNA expression for TRPV2 is greater than for TRPV4 and TRPV1 in rat distal pulmonary vein SMC and this expression was confirmed by Western blot [110]. However, in PA, TRPV2 expression is lower than that of TRPV4 [108,109]. TRPV2 is notably expressed in human and rat PASMC [108,112]. TRPV2 current shows a weakly outward rectifying I/V curve similar to the one observed in response to thromboxane A2 analogue. This suggests that TRPV2 is a potential candidate for non-selective cationic response to thromboxane A2. It is noteworthy that thromboxane A2 analogue has been shown to play a critical role in rat hypoxic pulmonary vasoconstriction, interfering that TRPV2 could also be implicated [112].

#### 3.2.3. TRPV4

TRPV4 is widely expressed in every layer of PA [109]. In PAEC, it plays a role in vasodilatation [113,114], whereas in PASMC it is involved in contraction, migration, and proliferation [9,65,108,115,116,117]; and in PAAF, it participates in remodeling processes [118]. All these mechanisms are either impaired (namely endothelial dysfunction) or enhanced (namely contraction and remodeling) in PH. Hence, TRPV4 activation in endothelium induces relaxation through NO-signaling and endothelium-derived hyperpolarizing factor [119]. Furthermore, TRPV4 interacts with eNOS in PAEC [114] and activation of unitary Ca^2+^ influx events through TRPV4 in these cells induces the release of NO, which initiates the guanylyl cyclase–protein kinase G pathway. Besides, guanylyl cyclase–protein kinase G signaling provides a negative feedback for TRPV4-eNOS coupling, thus regulating TRPV4 function [113]. Although TRPV4-induced Ca^2+^-signaling in PAEC is an important pathway for vasorelaxation, a dysregulation of this signaling is observed in PH. Indeed, in a rat model of PAH (SU5416 plus hypoxia) occurring through vascular endothelial growth factor receptor 2 (VEGFR2) inhibition, elevated production of mitochondrial-derived ROS induces a Ca^2+^ influx via TRPV4, which promotes enhanced migratory and proliferative responses in PAEC [120]. In PAAF, TRPV4 activation induces proliferation, migration, and extracellular matrix production (collagen I and fibronectin) thereby modulating the matrix stiffness, thus contributing to the adventitial remodeling occurring during PH [118]. PASMC express functional TRPV4 channels which are involved in increased [Ca^2+^]_i_, which triggers proliferation [111,115,117] and migration [107]. TRPV4 expression is increased in CH rat PASMC [23,65] associated with enhanced responses, providing evidence that TRPV4 is important in the development of the pathology. Indeed, the TRPV4 upregulation in PASMC is consistent with the higher basal [Ca^2+^]_i_ observed after CH, an enhanced myogenic tone and 5-HT-induced vasoconstriction via the cytochrome P450 epoxygenase–epoxyeicosatrienoic acid pathway CH PA [23,121]. Nevertheless, whereas culturing PASMC in CH (1% O_2_ during 48 h) does not affect TRPV4 expression, the amplitude of the TRPV4-induced Ca^2+^ elevation and migratory response are more elevated [21], suggesting a direct role of hypoxia on TRPV4 channel function. The deletion of the *trpv4* gene in mice has provided evidence of the implication of TRPV4 channel in the development of PH. Indeed, in CH *trpv4*^−/−^ mice, all the features of PH are reduced, such as right ventricle heart hypertrophy, remodeling via muscularization of the arteries [23], PA pressure increase associated with hypoxic pulmonary vasoconstriction [122], and enhancement in 5-HT induced contraction [116]. Moreover, in PASMC from IPAH patients, TRPV4 expression and shear stress-induced Ca^2+^ response are increased, whereas this hypersensitivity to mechanical stimuli is reduced by TRPV4 inhibition using pharmacological antagonists or siRNA knockdown [9].

### 3.3. TRPM

Among TRPM channels, TRPM3, TRPM4, and TRPM7 are known to be mechanosensitive [104]. TRPM3 is sensitive to hypotonic cell swelling [51] whereas TRPM4 implication in mechanosensitivity seems to be indirect [55,123]. TRPM7 has been described as sensitive to both stretch and osmolarity [58,124].

#### 3.3.1. TRPM3

TRPM3 mRNA expression in rat PA was shown by RT-qPCR [109]. Its expression is weaker than other TRPM channels such as TRPM8, TRPM4, and TRPM7 but higher than TRPM5 or TRPM6. However, no functional studies have been performed on TRPM3 in PA.

#### 3.3.2. TRPM4

TRPM4 is widely expressed in various tissues including SMC of rat aorta, cerebral arteries, and PA. In PA, TRPM4 mRNA expression is, quantitatively, the second most important TRPM channel detected after TRPM8 [109]. TRPM4 is selective for monovalent cations and has a calcium-dependent activation (with an EC_50_ of 10 µM in native SMC) [125]. The function of TRPM4 in PA has not been studied but it was shown that this channel is involved in the regulation of cerebral artery SMC membrane potential and contractility. Indeed, its activation induces Na^+^ influx in SMC under physiological conditions and a down-regulation of its expression impairs pressure-induced SMC depolarization and vasoconstriction in isolated rat cerebral arteries [55]. Another study reported that *trpm4*^−/−^ mice are hypertensive and present an increase in contractility of peripheral resistance vessels and cardiac function [126]. The role of TRPM4 in PH should thus be further investigated.

#### 3.3.3. TRPM7

Similar to the other TRPM channels, TRPM7 expression in rat PA has been evaluated by RT-qPCR and its mRNA levels are similar to those of TRPM4 [109]. TRPM7 is a divalent cation permeable ion channel with a greater permeability to Mg^2+^ than Ca^2+^ [56] and has been shown to contribute to various phenomena involved in PH such as inflammation, fibrosis, cell migration, and cell proliferation [127]. Several studies show its implication in enhanced proliferation and vascular remodeling associated with PH. Song et al. described the role of TRPM7 in sensing fluid flow shear stress in human PASMC from IPAH or normal patients. They also showed that TRPM7, as well as TRPV4, are necessary for shear stress-mediated Ca^2+^ increase in PASMC and that TRPM7 is also responsible for Mg^2+^ influx. Moreover, in IPAH PASMC, shear stress-induced Mg^2+^ increase is greater than in normal PASMC. This augmentation is due to upregulated TRPM7 expression in these cells [9]. Thus, upregulated mechanosensitive channels such as TRPM7 in PASMC may contribute to the development of IPAH by inducing sustained pulmonary vasoconstriction leading to vascular remodeling. Confirming these findings, a study showed the therapeutic effect of *Ophiocordyceps sinensis*, an entomopathogenic fungus, known to exert antiproliferative and antiremodeling effects on PH through TRPM7 inhibition. They found that TRPM7 is expressed in fibroblast-like cells of the right ventricle and in PA, and that this expression is increased in PH rats. TRPM7 deletion in PH mice attenuates the signs of PH. Inhibition of TRPM7 activity via this fungus has an antiproliferative effect in IPAH PASMC, and vaso-dilates human PA [59]. However, an opposite effect was observed in another study where, in condition of PH, TRPM7 currents and free Mg^2+^ concentration were reduced in human or rat PASMC. There, TRPM7 inhibition increased proliferation and apoptosis resistance in PASMC through MEK/ERK pathway and exacerbated hypoxia-induced PH in vivo, whereas TRPM7 overexpression decreased PASMC proliferation and apoptosis resistance [128].

### 3.4. TRPA1

TRPA1 is also mechanosensitive and proposed as a candidate in mechanically gated hair cell transduction in the auditory response [129], or as shown in Merkel cells from hamster buccal mucosa [105]. However, it has not been studied in pulmonary vessels.

### 3.5. TRPP

TRPP channels consist of TRPP1, TRPP2, and TRPP3. Among TRPP family, TRPP1 can interact with Polycystin-1 [60]; TRPP2 along with Polycystin-1, TRPV4, and TRPC1, then functioning as mechanosensitive channels [61]. TRPP1 and TRPP2 are both expressed in vascular smooth muscle and EC from cerebral and mesenteric arteries playing a role in regulation of blood vessel function and myogenic tone [130,131]. However, roles of TRPP channels in pulmonary vasculature are not known and require further investigation.

## 4. Piezo Channels

Ten years ago, the discovery of Piezo channels as new SAC by Coste et al. [132] opened up new research opportunities in the field of mechanotransduction. Firstly described in pressure sensitive neurons, their involvement in vascular physiopathology has emerged. Piezo are encoded by two genes, *Piezo1* and *Piezo2* (formerly named *Fam38A* and *Fam38B*), coding for two different channels: Piezo1 and Piezo2. Piezo channels have the particularity to form a homotrimeric protein complex. Hence, Piezo channels possess a propeller-shaped architecture that comprises the central cap and three peripheral blades. Each monomer presents 38 transmembrane domains [133] instead of the most common six transmembrane domains as for TRP channels. Piezo channels are mainly permeable to Ca^2+^ but also to Na^+^, K^+^, and Mg^2+^, therefore being non-selective Ca^2+^ channels [134] (Table 2). They are activated by several mechanical stress forces such as physiological shear stress [135], cell wall tension or, in vitro, via a membrane crushing by a pipette, positive, and negative pressures [132,136] or a hypo-osmotic stress. Recently, ultrasound waves (500 kHz) have also been shown to activate those channels [137]. Piezo1 and 2 have similar properties but Piezo1 appears to be preferentially activated by a negative pressure while Piezo2 by a positive one. The channel sensitivity to pressure also differs: compared to Piezo1, Piezo2 channel is activated by higher pressures [138]. Furthermore, selective chemical agonists for Piezo1 channel have been described: Yoda1, which acts as a gating modulator by sensitizing the activation threshold of the channel and slows its inactivation kinetic [139,140]; Jedi1/2, which modulates the channel activation by potentializing its mechanosensitivity and slows down its inactivation kinetic [141]. Several common antagonists of SAC also antagonize Piezo1 channel such as the GsMTx-4 toxin [142,143], Gd^3+^ or ruthenium red [132,144]. However, some selective antagonists of the Yoda1-induced Piezo1 activation have been described such as Dooku1 (a Yoda1 analogue) [145] or Tubeimoside I (TBMS1) [146].

Piezo1 channels have been implicated in the pathogenesis of several vascular diseases. Deletion of *Piezo1* gene in mouse leads to an aberrant vascular development resulting in an early embryonic death around day 10 [151]. In human, loss of function mutation of Piezo1 causes a congenital lymphatic dysplasia and patients are affected with a persistent lymphedema [152]. In systemic circulation, Piezo1 controls pressure or wall thickness [153,154]. Piezo1 channels display synergic or opposite functions in vessels, depending on the cell type where they are expressed. Endothelial Piezo1 channels are required for flow-induced vasodilatation due to eNOS activation and NO release [155]. Besides, the Ca^2+^ influx through these channels is not the only mechanism leading to NO formation as it also requires an ATP release through pannexin channels and subsequent P2Y2 receptor and endothelial NOS activation [155]. An alternative signaling pathway involves the release of adrenomedulin, which in turn, triggers NO release [156]. In uterine artery, as well as in the aorta, endothelial Piezo1 induces vessel relaxation [145,157]. By contrast, in mesenteric vessels, Piezo1 is responsible for flow-sensitive cationic ion influx in EC, which depolarizes the membrane. This depolarization spreads to the adjacent vascular SMC and activates voltage-gated Ca^2+^ channels causing vasoconstriction [158]. Retailleau et al. [153] reported that the increased cytosolic Ca^2+^ due to enhanced Piezo1 activation in vascular SMC influences both the diameter and wall thickness during hypertension in systemic circulation.

The presence of Piezo1 and 2 channels in the lung was described in the first report of Piezo as the tissue with the highest expression of these channels [132]. Others, as well as we, have reported the presence of Piezo1 in rat, mouse, or human PA [26,147,148,149]. Piezo2 is also present in human PA but its function remains unknown [147]. Human and mouse PAEC express Piezo1 channels [26]. The stimulation of this channel by a hypo-osmotic shock or by its selective agonist Yoda1 induces a large Ca^2+^ influx. The intracellular Ca^2+^ increase subsequently stimulates NO formation through eNOS, which brings about relaxation of mouse intrapulmonary arteries [26]. Unlike in the systemic circulation, Piezo1-induced NO formation does not require activation of ATP receptors. Selective deletion of Piezo1 in mouse EC inhibits Yoda-1-induced relaxation. Interestingly, endothelial deletion of Piezo1 increases α-adrenergic agonist-mediated contraction. Two hypotheses could explain this observation. On the one hand, phenylephrine-induced contraction of the vessel modifies its shape, which could lead to PAEC stretch, which, in turn, activates PAEC-Piezo1 channels. NO released by PAEC would then antagonize the contraction. On the other hand, phenylephrine could release factors derived from PASMC that may stimulate endothelial Piezo channels. In this connection, a crosstalk between SMC and endothelium has been previously described in pulmonary artery, where 5-HT stimulates both SMC contraction and NO release [159,160,161].

In CH mouse, Piezo1 still mediates arterial relaxation. Deletion of this channel in PAEC does not impair the development of the disease [26]. This result is supported by RT-qPCR measurements that show no variation in Piezo1 transcript in human PA from PH patients [147]. This finding is original as several other relaxation pathways are reduced during hypertension such as the acetylcholine pathway, which also requires NOS activation. As the Piezo1-mediated relaxation is still present in PH, activation of this channel could serve as therapeutic pathway.

In cultured human PASMC, Yoda1 induces a large intracellular Ca^2+^ increase due to activation of Piezo1 channels located at the plasma membrane and in intracellular organelles such as reticulum or mitochondria [149,162]. This Ca^2+^ rise through Piezo1 channels contributes to cell contraction and proliferation. In cultivated PASMC derived from IPAH patients, Piezo1 is upregulated and its activation triggers a higher Ca^2+^ influx (from both extracellular and intracellular Ca^2+^ stores) leading to an increased cell proliferation that could contribute to the pathophysiology of PH [149].

In conclusion, while endothelial Piezo1 function is not altered in mouse PA in PH, PASMC-Piezo1 channels are upregulated in human arteries [149], and could contribute to the development of the pathology. Furthermore, Piezo1 are also involved in several other pathophysiological processes in other tissues that share common pathological characteristics with PH. Indeed, activation of Piezo1 by hydrostatic pressure has been shown to activate inflammasome signaling pathways in macrophages [163] and in nucleus pulposus cells [164]. Turbulent flow induces Piezo1- and Gq/G11-mediated integrin activation, resulting in focal adhesion kinase-dependent NF-κB [165]. Those mechanisms could also contribute to PH and these pathways will also be interesting to explore in future studies. Moreover, the fact that Piezo1 activation is involved in free radical and NO production in PAEC [26] suggests that it could also contribute to the production of NOO^−^, a toxic free radical that has been shown to be involved in PH [166]. Finally, Piezo1 induces cell migration and metastasis in several cancers [167]. Furthermore, Piezo1 localizes at focal adhesions to activate integrin-FAK signaling [168]. As Piezo1 is mainly expressed in the PAEC lamellipodia [151], we could postulate that Piezo1 could be involved in the remodeling of PA during PH.

## 5. Other Mechanosensitive Channels

In contrast to cationic depolarizing channels such as TRPV4 and Piezo, stretch also activates K^+^ hyperpolarizing channels (Table 3) such as two-pore domain K^+^ channels (K2P: TREK-1, TREK-2, TRAAK), ATP-dependent K^+^ channels (K_ATP_) [169] or calcium-activated K^+^ channels (BK_Ca_) [170]. However, stretch is not the only activator of these channels as they mainly respond to physiological chemical activators. Their activation, inducing a cellular hyperpolarization, acts as counterparts to TRPV4 or Piezo1 depolarization activity, enabling fine-tuning of mechanosensation, reducing cellular contraction in response to mechanical forces [171]. Potassium flux is also involved in cell proliferation and inflammation [172,173].

Whereas expression of K2P (TREK-1 especially) channels in PA has been confirmed [180,181], involvement of these channels in PH has not been described yet. K_ATP_ channels have been shown to be expressed in rabbit and human PASMC where they are involved in the modulation of the resting membrane potential [178,179]. Furthermore, the K_ATP_ opener, Iptakalim, has been shown to prevent pulmonary resistance vascular remodeling, cell proliferation, endothelial dysfunction [182,183], and PH [184]. Early studies showed that BK_Ca_ channels were activated by stretch and arachidonic acid in rabbit PASMC [175]. Besides, their activation prevents the development of PH in rat [176,185]. However, the mechanosensitive part of the function of these K^+^ channels has not been studied in detail in PA cells and further investigation needs to be done.

## 6. Conclusions

**Figure 5:**
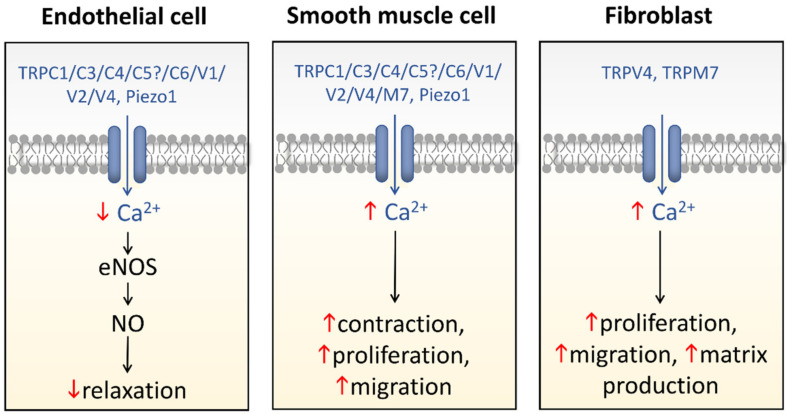
Schematic view illustrating the multifunctional contribution of SAC in the pathogenesis of PH. Red arrows indicate PH-induced modifications of cellular processes in pulmonary arterial vascular cells.

Mechanosensing is a key mechanism that intervenes in the regulation of the vascular tone. All the cellular layers of PA are able to sense mechanical forces, from PAEC to PASMC and PAAF. Each cell type responds differently and jointly to induce vasodilatation or vasocontraction, depending on the physiological needs and the sensed pressure. During PH, where mechanical stimulations in the PA are altered, modification of cellular processes occurs, mainly mediated through SAC (e.g., TRP and Piezo channels), see schematic Figure 5. Stretch-induced signaling pathways have been shown to disturb PAAF evoked-fibrosis, PAEC evoked-vasodilatation, PASMC evoked-contraction, and also their proliferation and migration. Altogether these alterations are involved in the pathophysiological phenotypes observed in PH. Future studies should aim to elucidate, in more details, mechanistic pathways that involve SAC in PA with the objective to target SAC in a pre-clinical approach. However, caution must be taken as mechanosensing is a key cellular mechanism that should not be annihilated at any cost in the whole organism and organs.

## Figures and Tables

**Table 1:** Biophysical properties of TRP channels in pulmonary arterial vascular cells.

Channel	Cell Type (Species)	Conductance (pS)	Permeability	Activator	Inhibitor	PA Phenotype in KO Mice	References
TRPA1	-	9–16 53.1–62.8	-	osmolarity mustard oil	HC030031	not described in PA	[35]
TRPC1	human, mouse, and rat PA (PAEC and PASMC)	16	P_Ca_/P_Na_ < 10	stretch store depletion	2-APB Gd^3+^/La^3+^	reduced hyperreactivity, remodeling, and vasomotor tone	[36,37,38,39]
TRPC3	rat PA human PASMC	66	P_Ca_/P_Na_ = 1.6	stretch store depletion DAG and analogs	2-APB Gd^3+^/La^3+^	not described in PA	[36,37,38,39]
TRPC4	human, mouse, and rat PA (PAEC and PASMC)	17.5–41	P_Ca_/P_Na_ = 1.1–7	store depletion, arachidonic acid calmidazolium	2-APB SKF96365 Gd^3+^/La^3+^ niflumic acid DIDS	reduced vascular permeability (and remodeling in KO rats)	[40,41,42,43,44,45,46,47]
TRPC5	human, mouse, and rat PA human PASMC	64	P_Ca_/P_Na_ = 9	stretch hypotonocity roziglitazone	2-APB La^3+^	not described in PA	[36,38,39,48,49,50]
TRPC6	human, mouse, and rat PA (PAEC and PASMC)	28–37	P_Ca_/P_Na_ = 4–5	stretch hypotonicity store depletion DAG and analogs	2-APB SKF96365 Cd^2+^, La^3+^, Gd^3+^	reduced hyperreactivity, remodeling and vasomotor tone	[36,37,38,39]
TRPM3	rat PA	65–133	P_Ca_/P_Na_ = 1.5–2	hypotonicity		not described in PA	[51,52]
TRPM4	rat PA	24–25	Na^+^//K^+^ > Cs^+^ > Li^+^	stretch		not described in PA	[52,53,54,55]
TRPM7	rat PA human and rat PASMC	105 22–37	Zn^2+^ ≈ Ni^2+^ >> Ba^2+^ > Co^2+^ > Mg^2+^ ≥ Mn^2+^ ≥ Sr^2+^ ≥ Cd^2+^ ≥ Ca^2+^ P_Na_/P_Ca_ = 3	stretch osmolarity		not viable → conditional deletion: reduced remodeling and vasomotor tone	[52,56,57,58,59]
TRPP1	-	135–175	-	PKD1 stretch	-	not described in PA	[36,60]
TRPP2	-	177	P_Ca_/P_Na_ = 1–5	shear stress hypotonicity triptolide	Gd^3+^/La^3+^	not described in PA (conditional in smooth muscle cells)	[36,37,61,62,63]
TRPV1	human and rat PA (PAEC and PASMC)	35–80 (unitary) 143–144 (whole cell)	P_Ca_/P_Na_ = 10 (capsaicin-activated)	hypotonicity capsaicin 2-APB cannabidiol resiniferatoxin	capsazepine AMG9810 A784168 5’-Iodoresiniferatoxin	not described in PA	[36,52,64]
TRPV2	human and rat PASMC	-	P_Ca_/P_Na_ = 1–3	hypotonicity stretch 2-APB	tranilast	not described in PA	[36,52]
TRPV4	human, mouse, and rat PA (PAAF PAEC, PASMC)	30–90 (unitary) 452 ± 63 (whole cell)	P_Ca_/P_Na_ = 6–10	hypotonicity shear stress 4-αPDD GSK1016790A	HC607047 RN-1734 GSK2193874	reduced hyperreactivity, remodeling and vasomotor tone	[23,36,52,65,66]

**Table 2:** Biophysical properties of Piezo channels in pulmonary arterial vascular cells.

Channel	Cell Type (Species)	Conductance (pS)	Permeability	Activator	Inhibitor	PA Phenotype in KO Mice	References
Piezo1	human, mouse, and rat PA human and mouse PAEC human PASMC	22–30	Ca^2+^ > Na^+^, K^+^, Mg^2+^ P_Ca_/P_Cs_ = 2; P_Na_/P_Cs_ = 1.1; P_K_/P_Cs_ = 1.1; P_Mg_/P_Cs_ = 0.5	negative and positive pressures, shear stress, ultrasound waves, Yoda1, Jedi1/2	GsMTx-4 ruthenium red Gd^3+^ dooku1 tubeimoside I	not viable → endothelium specific conditional deletion: no effect	[26,132,134,135,137,138,139,140,141,142,144,145,146,147,148,149,150]
Piezo2	human PA	27–28	-	negative and positive pressures	GsMTx-4 ruthenium red Gd^3+^	not described in PA	[132,136,138,143,147]

**Table 3:** Biophysical properties of mechanosensitive K^+^ channels in pulmonary arterial vascular cells.

Channel	Cell Type (Species)	Conductance (pS)	Permeability	Activator	Inhibitor	PA Phenotype in KO Mice	References
BK_Ca_	rabbit and rat PASMC	273	K^+^	negative pressure, calcium	-	not described in PA	[170,174,175,176]
K_ATP_	human and rabbit PASMC	42–55 (pressure), 28 (Levcromakalim)	K^+^	negative and positive pressures, levcromakalim, iptakalim	intracellular ATP glibenclamide	not described in PA	[177,178,179]
TREK-1	mouse PA rat PASMC	90	K^+^	negative pressure	-	not described in PA	[180,181]
TREK-2	mouse and rat PA	-	K^+^	stretch	-	not described in PA	[180,181]
